# Yiqi Huoxue Recipe Regulates Autophagy through Degradation of Advanced Glycation End Products via mTOR/S6K1/LC3 Pathway in Diabetic Nephropathy

**DOI:** 10.1155/2021/9942678

**Published:** 2021-08-09

**Authors:** Liang Chen, Linghao Dai, Yi Liu, Xinyue Li, Hui Wang

**Affiliations:** ^1^Laboratory and Facilities Management Office, Zhejiang Chinese Medical University, Hangzhou, Zhejiang 310053, China; ^2^College of Pharmacy, Zhejiang Chinese Medical University, Hangzhou, Zhejiang 310053, China

## Abstract

**Background:**

Yiqi Huoxue recipe can delay the progression of diabetic nephropathy, but its treatment mechanism is still unclear. We aimed to explore the effects of Yiqi Huoxue recipe on autophagy in diabetic nephropathy and its underlying mechanism.

**Methods:**

All rats were randomly divided into seven groups. The body weight, kidney weight, blood glucose, glycated hemoglobin, urine protein, urine microprotein, creatinine, urea nitrogen, triglyceride, and lipoprotein were analyzed. HE, Masson, and periodic acid-Schiff staining were used to detect the severity of pathological changes in kidneys. The level of advanced glycation end products was assessed by the ELISA. Immunofluorescence staining was performed to check the expressions of podocin and nephrin. The expression levels of mTOR/S6K1/LC3 pathway-related proteins and mRNA were detected by qRT-PCR and western blotting.

**Results:**

Yiqi Huoxue recipe significantly elevated body weight and significantly decreased kidney weight and kidney index. Yiqi Huoxue recipe significantly affected the levels of biochemical indicators in diabetic nephropathy and showed a regulatory effect on kidney damage and lipid metabolism disorders. ELISA showed that Yiqi Huoxue recipe significantly reduced the level of advanced glycation end products. The expressions of nephrin and podocin increased significantly, depending on the dosage of Yiqi Huoxue recipe. Additionally, Yiqi Huoxue recipe regulated the expression levels of p-AKT, mTOR, S6K1, and LC3.

**Conclusion:**

Yiqi Huoxue recipe regulates podocyte autophagy to promote the degradation of advanced glycation end products through mTOR/S6K1/LC3 pathway. It has a certain guiding significance for the diagnosis and treatment of diabetic nephropathy.

## 1. Introduction

Diabetic nephropathy (DN) is one of the serious complications of diabetes, occurring in about 40% of people with diabetes [1]. By 2045, diabetes patients are expected to reach 629 million [2, 3]. Moreover, the incidence of diabetes is increasing rapidly in China, causing a heavy social and economic burden. The clinical characteristics of DN are proteinuria, hypertension, renal dysfunction, and end-stage renal disease [4]. Pathogenesis of DN include podocyte injury and apoptosis, advanced glycation end products (AGEs) formation, production of reactive oxygen species (ROS), initiation of the polyol pathway, and stimulation of protein kinase C and the renin-angiotensin system [5, 6]. Drugs for the treatment of DN include benazepril hydrochloride, metformin, valsartan, and dapagliflozin. Benazepril hydrochloride is a commonly used drug in the clinical treatment of DN. It not only lowers blood pressure but also reduces the proteinuria level [7]. Traditional Chinese medicine also has provided effective treatment for DN [8]. However, the therapeutic mechanism of DN remains unclear.

Podocyte is one of the important components of renal tubular capillary filtration; its loss and damage are related to the occurrence and development of DN. Nephrin and podocin are two important podocyte-related protein molecules, which are mainly expressed and function in the glomerular filtration system [9]. Increasing evidence indicates that podocyte injury and apoptosis are involved in multiple signaling pathways, such as mTOR, Wnt/*β*-catenin, rho/GTP, and endoplasmic reticulum stress-related pathways [6]. Autophagy can maintain the normal structure and function of podocytes. Recent studies have shown that inhibition of podocyte autophagy leads to diabetic renal insufficiency [10]. The mTOR signaling pathway is not only an important factor regulating the growth and proliferation of podocyte but also a key regulator factor in the initiation of autophagy [11]. The activation of mTOR was observed in DN patients and mouse models, accompanied by decreased autophagy of podocytes [12]. The activation of mTOR leads to the expression of ribosomal S6 kinase 1 (S6K1) and LC3, thereby stimulating podocyte injury [13]. Pei et al. found that NAMPT promotes neuronal survival through inducing autophagic protein LC3II via regulating the TSC2/mTOR/S6K1 signaling pathway in a SIRT1-dependent manner during cerebral ischemia [14]. In DN rats, rapamycin affected podocyte autophagy through mTOR/S6K1/LC3II signaling pathway [4]. However, the detailed mechanism of the mTOR-mediated S6K1/LC3 pathway remains unknown.

In this study, we observed the effects of benazepril hydrochloride and different doses of Yiqi Huoxue recipe (YHR) on DN rats. The possible mechanisms of YHR in the autophagy of podocytes were also explored. Our findings suggested that YHR regulates podocyte autophagy to mediate the degradation of AGEs through the mTOR/S6K1/LC3 pathway in DN rats. It may provide a new direction for the clinical treatment of DN.

## 2. Methods

### 2.1. Rats

A total of 70 Sprague Dawley male rats weighing 150–180 g were obtained from the Experimental Animal Center. All rats were randomly divided into the normal control group (*n* = 8) and model group (*n* = 62). The model group was treated with high-fat and high-sugar foods consisting of 66.5% common feed, 20% sucrose, 10% lard, 2.5% cholesterol, and 1% pig bile salt for three weeks. Then, rats in the model group were intraperitoneally injected with streptozotocin (35 mg/kg) to induce the establishment of a diabetic rat model. Rats with random blood glucose higher than 16.7 mmol/L after 72 h were established as successful diabetes model rats. Rats in the control group were given an injection with citrate buffer. Based on the diabetes model rats, high-fat and high-sugar foods, short-acting insulin (20 U/kg, 06 : 00, 12 : 00, 18 : 00), and glucose (3 g/kg, 9 : 00, 15 : 00, 21 : 00) were given to result in a glucose fluctuation model. YHR was composed of *Astragalus* (20 g), *Codonopsis* (15 g), *Ligusticum chuanxiong* (20 g), leech powder (1 g), *Poria* (10 g), *Atractylodes* (10 g), Chinese yam (10 g), *Anemarrhena* (10 g), Habitat (10 g), *Cornus* (10 g), *Trichosanthes* (10 g), *Angelica* (10 g), red peony (10 g), *Salvia* (10 g), and roasted licorice (6 g). According to the equivalent conversion of the dose between human and rat, YHR and benazepril hydrochloride were given by gavage once a day. The duration of the drug treatments was 10 weeks. Glucose fluctuation rats were divided into 5 groups: blood glucose fluctuation group, benazepril hydrochloride group (0.9 mg/kg/d), YHR low-dose group, YHR middle-dose group, and YHR high-dose group. The workflow of this study is displayed in Supplemental Figure S1. The animal study was performed in Hangzhou Eyong Biotechnological Co., Ltd. Animal Experiment Center (certificate no. SYXK (Zhe)2020-0024), and the ethical code is EYOUNG-20201117-02.

At the 16^th^ weekend, the body weights of the rats were weighted with an electronic balance (UTP-313, Hochoice, Shanghai, China). The rats fasted for 12 h and blood was collected from the abdominal aorta. Then, rats were dissected and kidneys were extracted. The upper serum was collected by centrifuge at 3000 rpm/min for 15 min and stored at −20°C. The blood glucose, glycosylated hemoglobin, urine protein, urine microprotein, creatinine, urea nitrogen, triglyceride (TG), low-density lipoprotein (LDL), and high-density lipoprotein (HDL) were measured by automatic biochemical analyzer (C16000, Abbott Laboratories, Chicago, USA) within 48 h.

### 2.2. UPLC-Q/TOF-MS Analysis

The chemical components of YHR were assessed by Waters ACQUITYI-Class Plus Ultra Performance Liquid Chromatography (UPLC) system (Waters, USA) and SCIEX X-500R four-stage rod time-of-flight mass spectrometer (AB SCIEX, USA). SCIEX OS software was used to collect and manage data.

### 2.3. Measurement of AGEs

The contents of AGEs in serum and kidney tissue were assessed using enzyme-linked immunosorbent assay (ELISA) kits (MM-0776R1, MEIMIAN, Jiangsu, China). First, we diluted the 20-fold concentrated washing liquid with distilled water and set standard wells (50 *μ*L standard substance + 100 *μ*L HRP-labeled antibody), test wells (10 *μ*L sample + 40 *μ*L diluent + 100 *μ*L HRP-labeled antibody), and control wells. Then, we sealed the reaction wells and incubated them in a water bath for 60 min. After washing, we added 50 *μ*L A solution and 50 *μ*L B solution. In the end, 50 *μ*L solution was used to stop the reaction and the absorbance of each well was measured.

### 2.4. Histological Analysis

Kidneys were placed in 4% paraformaldehyde solution for 48 h at 4°C. The 4 *μ*m sections were cut from paraffin-embedded samples and were stored at normal temperature. Sections were stained with HE, Masson (G1006, ServiceBio, Wuhan, China), and periodic acid-Schiff (PAS, G1281, Solarbio, Beijing, China). The details of HE staining were as follows: (1) the slices were routinely deparaffinized with xylene and washed with ethanol and water; (2) stained by hematoxylin for 5 min; (3) differentiated in hydrochloric acid ethanol solution for 30 s; (4) put it in eosin solution for 2 min and rinsed with running water; and (5) dehydrated and sealed. The severity of pathological changes of kidney was scaled from 0 to 3. The higher the score, the more severe the kidney damage. Masson's trichrome reagent was analyzed by NIS-Elements 3.2 software (Nikon, Tokyo, Japan). Then, we used microscope (DM3000, Leica, Germany) to capture and analyze image.

### 2.5. Immunofluorescence Staining

The paraffin sections were dewaxed with xylene and ethanol, and then we placed the sections in EDTA buffer for antigen repair. After blocked with antinephrin (sc-376522, Santa Cruz, USA) and antipodocin primary antibody (sc-518088, Santa Cruz, USA) at 4°C overnight, we washed the sections with PBS for 3 times and incubated them with goat anti-mouse IgG (ab150113, Abcam, Cambridge, UK) at room temperature for 50 min. Then, we stained the nucleus with DAPI (C1002, Beyotime, Shanghai, China) and sealed the sections with antifluorescent quench sealant (P0126, Beyotime, Shanghai, China). At last, the sections were observed under an inverted fluorescence microscope (Nikon, Japan).

### 2.6. qRT-PCR

The primer sequences used in this study are shown in Supplemental Table S1. We performed RNA extraction in homogenate tubes containing 1000 *µ*l TRIzol and 200 mg tissue. Chloroform and isopropanol were subsequently added to the supernatant, and the mixture was centrifuged at 12000 rpm for 10–15 min. After the supernatant was discarded, we added 75% anhydrous ethanol to rinse the precipitation 2 times. Then, we used DEPC (diethyl pyrocarbonate) water to dissolve RNA and store it at −80°C. The reverse transcription kit (CW2569, CWBIO, China) was used in our study. The condition of reverse transcription reaction was as follows: 42°C, 15 min, and 85°C, 5 min. The components were 4 *μ*L dNTP Mix, 2 *μ*L primer mix, 1 *μ*L RNA template, 4 *μ*L 5xRT buffer, 2 *μ*L DTT, 1 *μ*L HiFiScript, and 20 *μ*L RNase-free double-distilled H_2_O. Real-time fluorescent quantitative PCR system consisted of 10 *μ*L 2xUltraSYBR mixture, 1 *μ*L PCR forward primer, 1 *μ*L PCR reverse primer, 2 *μ*L cDNA template, 6 *μ*L double-distilled H_2_O, and the condition was denaturation at 95°C for 10 min; 95°C, 15 s; and 60°C, 60 s, 40-time cycle.

### 2.7. Western Blotting

First, we added 600 *µ*L RIPA lysis buffer (P0013D, Beyotime, Shanghai, China) with phenylmethanesulfonyl fluoride (PMSF) and protease inhibitor into cells. The total protein concentration of samples was determined using the BCA protein assay kit (pc0020, Solarbio, Beijing, China). The sample (20 *µ*g) was separated by 10% SDS-PAGE and transferred to PVDF membrane (10600023, GE Healthcare Life, USA). After the membrane was washed with TBST 3 times, we blocked the PVDF membrane in 5% skim milk for 1.5–2 h. Then, we incubated the membrane with the primary antibody (anti-AKT: Abcam, ab8805, 1 : 500; anti-p-AKT: Abcam, ab38449, 1 : 1000; mTOR antibody: Affinity, AF6308, 1 : 1000; anti-S6K1: Abcam, ab32359, 1 : 1000; anti-LC3: Abcam, ab51520, 1 : 3000; and anti-beta actin: Abcam, ab8227, 1 : 4000) and secondary antibody (anti-rabbit IgG, HRP-linked antibody: CST, 7074, 1 : 3000; anti-mouse IgG, HRP-linked antibody: CST, 7076, 1 : 3000), separately. At last, the PVDF membrane was incubated with ECL luminescence reagent and analyzed with Chemi capture software.

### 2.8. Statistical Analysis

Data analysis was performed using SPSS 16.0 software (SPSS Inc., USA). All data were expressed as the mean ± SD. *P* < 0.05 was regarded as significant difference. Between two groups, a two-sample independent *t*-test was used for homogeneity of variance, and Kruskal–Wallis *H* test was used for heterogeneity of variance.

## 3. Results

### 3.1. The Chemical Components of Yiqi Huoxue Recipe

According to the analysis of the UHPLC-Q/TOF-MS system, the total ion current was presented in Supplemental Figure S2. The main components of YHR were puerarin, ferulic acid, caffeic acid, and astragaloside I by comparing and screening with the secondary database of Chinese medicine in SCIEX OS software (Figure S3). There were 39 and 38 chromatographic peaks assigned to the positive ion mode and negative ion mode, individually (Tables S2 and S3).

### 3.2. Yiqi Huoxue Recipe Elevates Body Weight and Alleviates Kidney Dysfunction of Diabetic Nephropathy Rats

**Figure 1:**
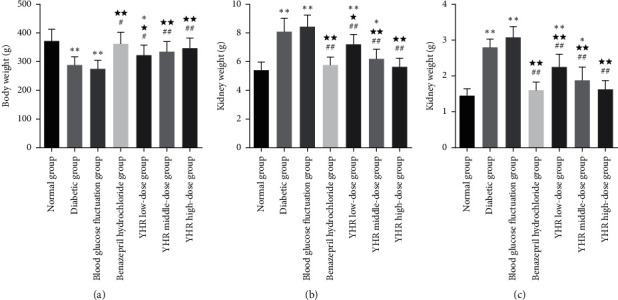
The effects of Yiqi Huoxue recipe on signs of rats. (a) Body weight. (b) Kidney weight. (c) Kidney index. Compared with the normal group, ^*∗*^*P* < 0.05, ^*∗∗*^*P* < 0.01. Compared with the diabetic group, ^★^*P* < 0.05, ^★★^*P* < 0.01. Compared with blood glucose fluctuation group, ^#^*P* < 0.05, ^##^*P* < 0.01.

As shown in Figure 1 and Table 1, YHR significantly elevated body weight and decreased kidney weight and kidney index (*P* < 0.05). Compared with the normal group, the body weight decreased significantly (*P* < 0.01), whereas kidney weight and kidney index increased significantly (*P* < 0.01) in the diabetic group, blood glucose fluctuation group, and YHR low-dose group. For rats in the YHR high-dose group and the benazepril hydrochloride group, no significant differences were observed in body weight, kidney weight, and kidney index (*P* > 0.05). Compared with the diabetic group or blood glucose fluctuation group, the body weights of rats were significantly increased, and kidney weight and kidney index had declined significantly after the administration of benazepril hydrochloride and YHR, which was in a dose-dependent relationship.

### 3.3. The Effects of Yiqi Huoxue Recipe on Serum Biochemical Indicators in Diabetic Nephropathy Rats

**Figure 2:**
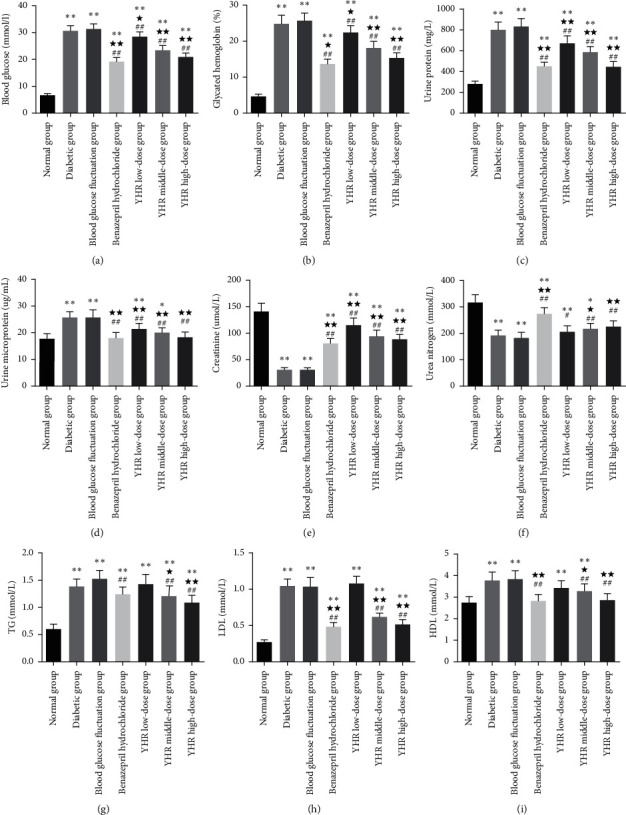
The effects of Yiqi Huoxue recipe on serum biochemical indicators of rats. (a) Blood glucose. (b) Glycated hemoglobin. (c) Urine protein. (d) Urine microprotein. (e) Creatinine. (f) Urea nitrogen. (g) TG: triglyceride. (h) LDL: low-density lipoprotein. (i) HDL: high-density lipoprotein. Compared with the normal group, ^*∗*^*P* < 0.05, ^*∗∗*^*P* < 0.01. Compared with the diabetic group, ^★^*P* < 0.05, ^★★^*P* < 0.01. Compared with the blood glucose fluctuation group, ^#^*P* < 0.05, ^##^*P* < 0.01.

In Figure 2 and Table 2, we found that YHR has a significant impact on the levels of blood glucose, glycated hemoglobin, urine protein, urine microprotein, creatinine, urea nitrogen, triglyceride (TG), low-density lipoprotein (LDL), and high-density lipoprotein (HDL) (*P* < 0.05). Compared with the normal group, the contents of blood glucose, glycated hemoglobin, urine protein, urine microprotein, TG, LDL, and HDL significantly increased (*P* < 0.01), whereas creatinine and urea nitrogen decreased in the diabetic group, blood glucose fluctuation group, YHR low-dose group, and YHR middle-dose group (*P* < 0.01). YHR treatment efficiently reduced the elevations of these biochemical indicators, and the effect was dose-dependent. Meanwhile, YHR induced a rise in creatinine and urea nitrogen (*P* < 0.05). These results indicate that YHR exhibits protective effects on kidney injury and lipid metabolism disorder in diabetic rats.

### 3.4. The Effects of Yiqi Huoxue Recipe on Advanced Glycation End Products

AGEs levels were significantly increased in the diabetic group and blood glucose fluctuation group compared to controls (*P* < 0.01, Table 3). In kidney homogenate, the contents of AGEs were significantly decreased in the benazepril hydrochloride group, YHR low-dose group, YHR middle-dose group, and YHR high-dose group compared with the diabetic group and blood glucose fluctuation group. YHR treatment also resulted in a significant reduction of tissue-AGEs in comparison with the diabetic group and blood glucose fluctuation group.

### 3.5. The Effects of Yiqi Huoxue Recipe on Pathological Morphology of Kidney Tissue

**Figure 3:**
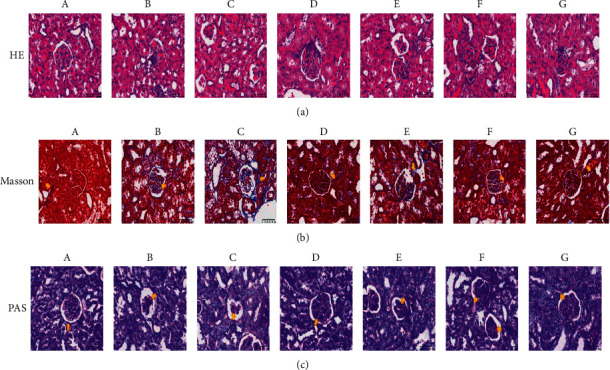
(a) HE, (b): Masson, and (c) Periodic Acid-Schiff staining (400 X). A: normal group; B: diabetic group; C: blood glucose fluctuation group; D: benazepril hydrochloride group; E: YHR low-dose group; F: YHR middle-dose group; G: YHR high-dose group.

HE, Masson, and PAS staining were used to observe the pathological changes of kidney tissue in rats. As presented in Table 4, the HE semiquantitative scores of kidney tissue in the administered group were significantly decreased compared with the model group (*P* < 0.05). In Figure 3, HE staining showed diabetic rats and rats with blood glucose fluctuations had enlarged renal capsule, focal degeneration, and atrophy of renal tubules, slightly thickened glomerular basement membrane, and mesangial matrix hyperplasia, and the glomerulus and renal tubules have fatty degeneration compared with the normal rats. After administration, the lesions were reduced and the improvement was more obvious in the YHR high-dose group. Masson staining indicated that the renal capsules and glomerular mesangial area were enlarged, blue fibrous deposits were seen in the glomerulus, and the mesangial matrix proliferated in the model group. After the treatment of benazepril hydrochloride and YHR, the blue fiber deposition was significantly decreased. The results of PAS staining were similar to those of HE and Masson. These findings suggested that YHR improved the pathological changes in the kidney of diabetic rats.

### 3.6. The Expression Levels of Podocin and Nephrin Detected by Immunofluorescence Staining

**Figure 4:**
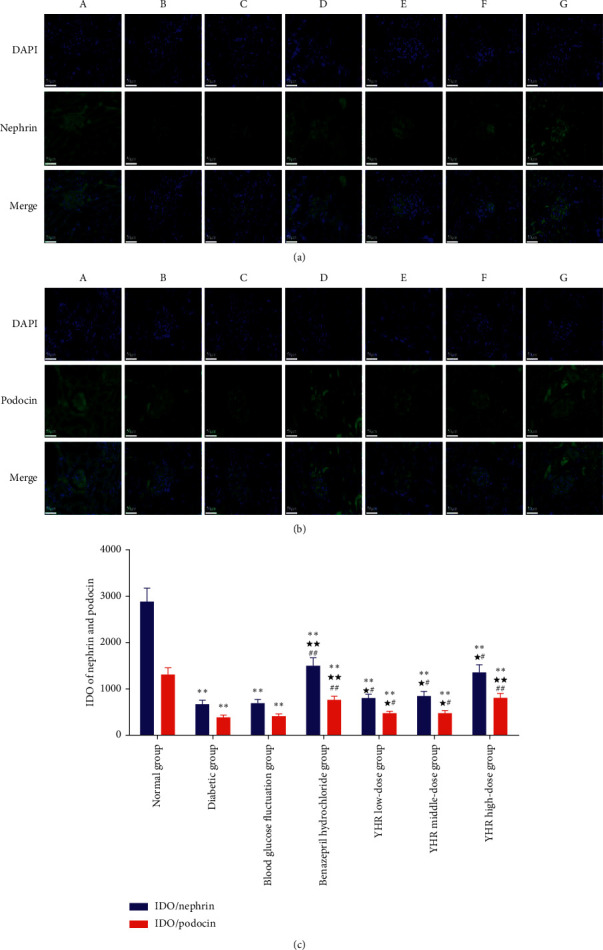
The effects of Yiqi Huoxue recipe on podocyte autophagy of rats (200 X). (a) Immunofluorescence staining of nephrin (blue color: DAPI, green color: nephrin). (b) Immunofluorescence staining of podocin (blue color: DAPI, green color: Podocin). (c) IDO of nephrin and podocin. A: normal group; B: diabetic group; C: blood glucose fluctuation group; D: benazepril hydrochloride group; E: YHR low-dose group; F: YHR middle-dose group; G: YHR high-dose group. Compared with the normal group, ^*∗*^*P* < 0.05, ^*∗∗*^*P* < 0.01. Compared with the diabetic group, ^★^*P* < 0.05, ^★★^*P* < 0.01. Compared with the blood glucose fluctuation group, ^#^*P* < 0.05, ^##^*P* < 0.01.

As shown in Figures 4(a)–4(c), there were significant differences in podocin and nephrin expression in the kidney tissues of rats from the seven groups. Compared with the normal group, the expressions of nephrin and podocin in the diabetic group and blood glucose fluctuation group were significantly reduced (*P* < 0.01). There was no significant difference in the expression of nephrin and podocin between the diabetic group and blood glucose fluctuation. Compared with the blood glucose fluctuation group, the expressions of nephrin and podocin in the administration group increased to varying degrees.

### 3.7. Yiqi Huoxue Recipe Regulated the mTOR/S6K1/LC3 Signaling Pathway

**Figure 5:**
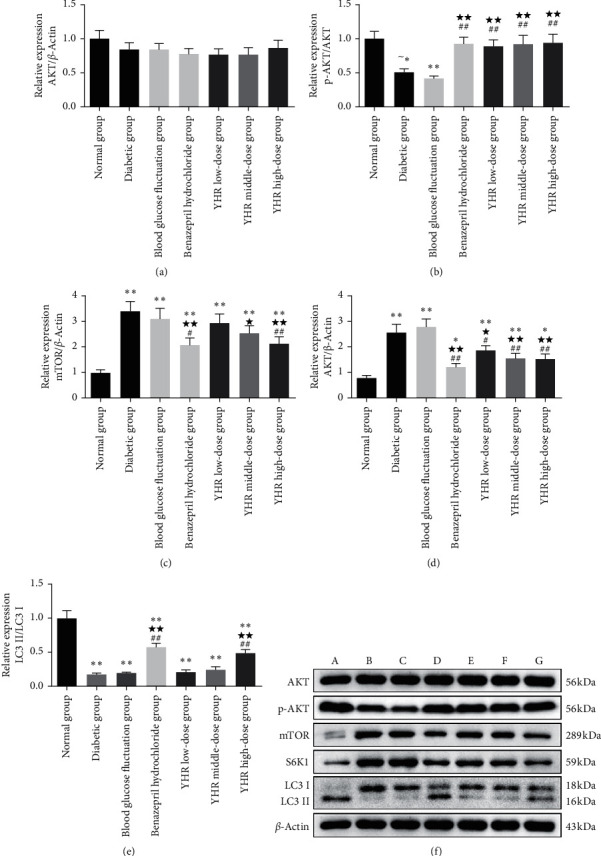
The expressions of Akt, p-Akt, mTOR, S6K1, LC3I, and LC3II in the kidney tissues of rats detected by western blotting. The ratio of LC3II/LC3I represents the level of autophagy. Compared with the normal group, ^*∗*^*P* < 0.05, ^*∗∗*^*P* < 0.01. Compared with the diabetic group, ^★^*P* < 0.05, ^★★^*P* < 0.01. Compared with blood glucose fluctuation group, ^#^*P* < 0.05, ^##^*P* < 0.01.

Table 5, Figure 5, and Figure S4 show the effects of YHR on autophagy by regulating the mTOR/S6K1/LC3 signaling pathway. Compared with the normal group, the mRNA expressions of mTOR and S6K1 were significantly increased in the diabetic group, blood glucose fluctuation group, benazepril hydrochloride group, YHR middle-dose group, and YHR high-dose group (*P* < 0.01), whereas LC3 II mRNA expression significantly decreased. The treatment of benazepril hydrochloride and YHR effectively reversed the mRNA expression of mTOR, S6K1, and LC3 II (*P* < 0.05). Western blotting results showed that the protein expression levels of p-AKT and LC3 were significantly decreased, and mTOR and S6K1 were significantly increased in the diabetic group and blood glucose fluctuation group compared with the normal group. After treatment with benazepril hydrochloride and high-dose YHR, the protein levels of p-AKT and LC3 were remarkably increased (*P* < 0.01), and mTOR and S6K1 protein expressions were significantly decreased.

## 4. Discussion

This study showed that YHR could significantly improve kidney damage of DN rats, which may be caused by increased expressions of nephrin and podocin in podocytes and AGEs degradation. Moreover, YHR induced autophagy of podocytes via regulating the mTOR/S6K1/LC3 signaling pathway. Thus, our results indicated that YHR might play a protective role by accelerating the autophagy of podocytes.

YHR is based on nourishing qi and promoting blood circulation to resolve blood stasis. The main compounds of YHR were puerarin, ferulic acid, caffeic acid, and astragaloside I. Li et al. found that puerarin could protect podocytes from diabetes-induced injury through HMOX1 and Sirt1-mediated upregulation of autophagy [15]. Ferulic acid could improve podocyte injury in DN rats by attenuating oxidative stress, inflammation, and fibrosis [16]. It suggests that the therapeutic effects of YHR on DN rats were realized through puerarin, ferulic acid, caffeic acid, and so on. YHR can contribute to the treatment of DN and improve the clinical symptoms of the disease, including hyperphagia, polydipsia, polyuria, weight loss, and blood glucose levels [17]. Wu et al. reported that YHR can reduce the levels of Th1 and Th2 related cytokines and the ratio of interferon-gamma/IL-4 in serum and submaxillary glands of nonobese diabetic mice with Sjogren's syndrome [18]. A recent meta-analysis of randomized controlled trials showed that YHR had positive effects on DN patients [19]. In this study, we observed that YHR elevated body weight and improved kidney function in DN rats, which is dependent on the dosage of medication. YHR also significantly affected the levels of TG, LDL, and HDL, exhibiting improvement of lipid metabolism disorder in DN. It is consistent with previous studies.

Autophagy is a highly regulated lysosomal pathway involved in cytoplasmic circulation and clearance of redundant or damaged organelles, which is essential for cell survival, differentiation, development, and homeostasis [20]. Nutrient-sensing pathways are considered to be effective regulators of autophagy, including mTOR, AMPK, and Sirt1 [21]. The basic autophagy activity of podocytes is highly constitutive, suggesting that the autophagosomal system plays a key role in maintaining podocyte homeostasis [22]. Previous studies showed that induction of autophagy may be a potential method to alleviate podocyte injury and treat DN [23]. Inhibition of autophagy results in podocyte damage mainly through the AKT/mTOR pathway [24]. Lei et al. reported that rapamycin has a protective effect on autophagy in renal tissues of DN rats via the mTOR/S6K1/LC3II signaling pathway [4]. In this study, we found that YHR plays a role in treating DN by regulating podocyte autophagy. Moreover, YHR specially regulated the expression levels of mTOR, S6K1, and LC3, indicating the effect of YHR on autophagy was related to the mTOR/S6K1/LC3 signaling pathway.

AGEs are proteins or lipids that are nonenzymatically glycosylated or oxidized after translation, and high levels of AGEs are related to activation of proinflammation pathways and contribute to aseptic inflammation [25]. AGEs could activate NF-*κ*B signaling cascade through directly binding to RAGEs or indirectly inducing ROS production. Zabad et al. presented that vanillin is beneficial for the prevention of DN by decreasing AGEs formation [1]. Additionally, results showed that autophagy is involved in the degradation of AGEs in DN by upregulating the biogenesis and function of lysosomes [26]. Similarly, our study indicated that YHR significantly decreased AGEs levels, which might be due to the regulation of podocyte autophagy.

Hence, this study firstly explored that the effects of YHR on DN rats and its potential mechanisms. These findings revealed that YHR has a protective effect on DN rats. The possible mechanism is that YHR regulates podocyte autophagy through the mTOR/S6K1/LC3 pathway and mediates the degradation of AGEs in DN.

## 5. Conclusion

In conclusion, this study indicated that YHR promotes podocyte autophagy and decreases the levels of AGEs by regulating the mTOR/S6K1/LC3 signaling pathway. These findings strengthen the theoretical basis of YHR in DN therapy and provide a potential target for further study on the pathogenesis and treatment of DN.

## Figures and Tables

**Table 1:** Body indicators of rats in each group ($\overset{‾}{\chi }$, *n* = 8).

Group	Body weight (g)	Kidney weight (g)	Kidney index (%)
Normal group	373.88 ± 38.39	5.42 ± 0.57	1.46 ± 0.19
Diabetic group	288.51 ± 29.11^*∗∗*^	8.11 ± 0.92^*∗∗*^	2.82 ± 0.21^*∗∗*^
Blood glucose fluctuation group	275.68 ± 29.02^*∗∗*^	8.46 ± 0.80^*∗∗*^	3.08 ± 0.30^*∗∗*^
Benazepril hydrochloride group	361.28 ± 40.11^☆☆#^	5.76 ± 0.57^☆☆##^	1.61 ± 0.22^☆☆##^
YHR low-dose group	322.64 ± 34.08^*∗*^^☆#^	7.21 ± 0.69^*∗∗*^^☆##^	2.26 ± 0.34^*∗∗*^^☆☆##^
YHR middle-dose group	335.64 ± 33.91^☆☆##^	6.22 ± 0.67^*∗*^^☆☆##^	1.88 ± 0.36^*∗*^^☆☆##^
YHR high-dose group	347.76 ± 35.02^☆☆##^	5.65 ± 0.60^☆☆##^	1.64 ± 0.23^☆☆##^

YHR: Yiqi Huoxue recipe. Compared with the normal group, ^*∗*^*P* < 0.05, ^*∗∗*^*P* < 0.01. Compared with the diabetic group, ^☆^*P* < 0.05, ^☆☆^*P* < 0.01. Compared with the blood glucose fluctuation group, ^#^*P* < 0.05, ^##^*P* < 0.01.

**Table 2:** Biochemical indicators of rats in each group ($\overset{‾}{\chi }$ ± *s, n* = 8).

Group	Blood glucose (mmol/L)	Glycated hemoglobin (%)	Urine protein (mg/L)	Urine microprotein (ug/mL)	Creatinine (umol/L)	Urea nitrogen (mmol/L)	TG (mmol/L)	LDL (mmol/L)	HDL (mmol/L)
Normal group	6.78 ± 0.57	4.66 ± 0.61	279.49 ± 28.96	17.82 ± 1.80	141.55 ± 15.31	317.58 ± 28.37	0.61 ± 0.09	0.27 ± 0.04	2.75 ± 0.28
Diabetic group	30.63 ± 1.94^*∗∗*^	24.86 ± 2.26^*∗∗*^	806.46 ± 68.78^*∗∗*^	25.69 ± 2.26^*∗∗*^	31.17 ± 4.26^*∗∗*^	193.17 ± 19.40^*∗∗*^	1.38 ± 0.14^*∗∗*^	1.05 ± 0.10^*∗∗*^	3.78 ± 0.39^*∗∗*^
Blood glucose fluctuation group	31.41 ± 1.85^*∗∗*^	25.77 ± 2.09^*∗∗*^	832.47 ± 76.51^*∗∗*^	25.72 ± 2.89^*∗∗*^	31.30 ± 3.41^*∗∗*^	183.93 ± 19.05^*∗∗*^	1.53 ± 0.15^*∗∗*^	1.04 ± 0.12^*∗∗*^	3.83 ± 0.40^*∗∗*^
Benazepril hydrochloride group	19.33 ± 1.53^*∗∗*^^☆☆##^	13.63 ± 1.37^*∗∗*^^☆☆##^	450.02 ± 41.88^*∗∗*^^☆☆##^	18.15 ± 1.91^☆☆##^	81.32 ± 8.35^*∗∗*^^☆☆##^	274.13 ± 23.83^*∗∗*^^☆☆##^	1.24 ± 0.13^*∗∗*^^##^	0.48 ± 0.06^*∗∗*^^☆☆##^	2.82 ± 0.29^☆☆##^
YHR low-dose group	28.59 ± 1.72^*∗∗*^^☆##^	22.40 ± 1.90^*∗∗*^^☆##^	674.48 ± 68.27^*∗∗*^^☆☆##^	21.45 ± 2.01^*∗∗*^^☆☆##^	115.97 ± 13.05^*∗∗*^^☆☆##^	206.38 ± 21.49^*∗∗*^^#^	1.43 ± 0.17^*∗∗*^	1.08 ± 0.10^*∗∗*^	3.45 ± 0.34^*∗∗*^
YHR middle-dose group	23.41 ± 1.86^*∗∗*^^☆☆##^	18.11 ± 1.81^*∗∗*^^☆☆##^	585.84 ± 53.60^*∗∗*^^☆☆##^	20.00 ± 1.77^*∗*^^☆☆##^	94.83 ± 10.42^*∗∗*^^☆☆##^	216.45 ± 21.73^*∗*^^★##^	1.21 ± 0.19^*∗∗*^^☆##^	0.62 ± 0.05^*∗∗*^^☆☆##^	3.29 ± 0.32^*∗∗*^^★##^
YHR high-dose group	20.90 ± 1.56^*∗∗*^^☆☆##^	15.30 ± 1.43^*∗∗*^^☆☆##^	446.32 ± 49.28^*∗∗*^^☆☆##^	18.44 ± 1.90^☆☆##^	88.82 ± 8.66^*∗∗*^^☆☆##^	226.31 ± 21.05^☆☆##^	1.10 ± 0.12^*∗∗*^^☆☆##^	0.52 ± 0.06^*∗∗*^^☆☆##^	2.87 ± 0.29^☆☆##^

YHR: Yiqi Huoxue recipe; TG: triglyceride; LDL: low-density lipoprotein; HDL: high-density lipoprotein. Compared with the normal group, ^*∗*^*P* < 0.05, ^*∗∗*^*P* < 0.01. Compared with the diabetic group, ^☆^*P* < 0.05, ^☆☆^*P* < 0.01. Compared with the blood glucose fluctuation group, ^#^*P* < 0.05, ^##^*P* < 0.01.

**Table 3:** The contents of AGEs in blood and kidney tissues of rats (‾*χ* ± *s, n* = 8).

Group	Serum-AGEs (ng/ml)	Tissue-AGEs (ng/ml)
Normal group	0.64 ± 0.06	0.68 ± 0.06
Diabetic group	0.93 ± 0.10^*∗∗*^	0.93 ± 0.08^*∗∗*^
Blood glucose fluctuation group	0.95 ± 0.08^*∗∗*^	1.01 ± 0.10^*∗∗*^
Benazepril hydrochloride group	0.66 ± 0.08^☆☆##^	0.73 ± 0.09^☆☆##^
YHR low-dose group	0.82 ± 0.08^*∗∗*^^☆##^	0.85 ± 0.07^*∗∗*^^☆##^
YHR middle-dose group	0.81 ± 0.06^*∗∗*^^☆##^	0.78 ± 0.09^*∗*^^☆☆##^
YHR high-dose group	0.69 ± 0.08^☆☆##^	0.74 ± 0.06^☆☆##^

YHR: Yiqi Huoxue recipe; AGEs: advanced glycation end products. Compared with the normal group, ^*∗*^*P* < 0.05, ^*∗∗*^*P* < 0.05. Compared with the diabetic group, ^☆^*P* < 0.05, ^☆☆^*P* < 0.01. Compared with the blood glucose fluctuation group, ^#^*P* < 0.05, ^##^*P* < 0.01.

**Table 4:** HE semiquantitative scale of rats (‾*χ* ± *s, n* = 6).

Group	Semiquantitative scoring
Normal group	—
Diabetic group	2.83 ± 0.41
Blood glucose fluctuation group	2.67 ± 0.52
Benazepril hydrochloride group	1.17 ± 0.75^☆☆##^
YHR low-dose group	1.83 ± 0.75^☆#^
YHR middle-dose group	1.67 ± 0.82^☆#^
YHR high-dose group	1.50 ± 0.55^☆☆##^

YHR: Yiqi Huoxue recipe. 0: normal; 1: the glomerular basement membrane thickened slightly and the mesangial membrane proliferated slightly; 2: the renal capsule was significantly enlarged, the renal tubules were focal degeneration, the glomerular basement membrane was obviously thickened, and the mesangial membrane was hyperplasia; 3: the glomerular basement membrane was thickened and the mesangial membrane was hyperplasia. Compared with the diabetic group, ^☆^*P* < 0.05, ^☆☆^*P* < 0.01. Compared with blood glucose fluctuation group, ^#^*P* < 0.05, ^##^*P* < 0.01.

**Table 5:** The mTOR, S6K1, and LC3 II expression in the kidney tissues detected by qRT-PCR (‾*χ* ± *s*, *n* = 3).

Group	mTOR	S6K1	LC3 II
Normal group	1.00 ± 0.11	1.00 ± 0.11	1.00 ± 0.10
Diabetic group	4.83 ± 0.53^*∗∗*^	3.59 ± 0.41^*∗∗*^	0.43 ± 0.06^*∗∗*^
Blood glucose fluctuation group	5.02 ± 0.62^*∗∗*^	3.89 ± 0.46^*∗∗*^	0.47 ± 0.07^*∗∗*^
Benazepril hydrochloride group	2.64 ± 0.27^*∗∗*^^☆☆##^	2.07 ± 0.21^*∗∗*^^☆☆##^	0.65 ± 0.07^*∗∗*^^☆#^
YHR low-dose group	3.66 ± 0.40^*∗∗*^^☆#^	2.70 ± 0.29^*∗∗*^^☆#^	0.87 ± 0.09^☆☆##^
YHR middle-dose group	3.27 ± 0.36^*∗∗*^^☆#^	2.52 ± 0.27^*∗∗*^^☆#^	0.73 ± 0.08^*∗*^^☆☆#^
YHR high-dose group	2.86 ± 0.30^*∗∗*^^☆☆##^	2.20 ± 0.39^*∗∗*^^☆☆##^	0.72 ± 0.08^*∗*^^☆☆#^

YHR: Yiqi Huoxue recipe. Compared with the normal group, ^∗^*P* < 0.05, ^∗∗^*P* < 0.01. Compared with the diabetic group, ^☆^*P* < 0.05, ^☆☆^*P* < 0.01. Compared with the blood glucose fluctuation group, ^#^*P* < 0.05, ^##^*P* < 0.01.

## Data Availability

All data are available within the article.

## Supplementary Materials

Supplementary Materials Table S1: primers in this study. Table S2: the chemical composition of Yiqi Huoxue recipe under positive ion mode. Table S3: the chemical composition of Yiqi Huoxue recipe under negative ion mode. Figure S1: the workflow of this study. Figure S2: the total ion chromatogram of Yiqi Huoxue recipe detected by UHPLC-Q-TOF-MS. Figure S3: schematic diagram of TCM secondary spectrum library comparison with TCM MS/MS Library. Figure 4: the mTOR, S6K1, and LC3 II expression in the kidney tissues detected by qRT-PCR.
