# Supplementary material for: Yiqi Huoxue Recipe Regulates Autophagy through Degradation of Advanced Glycation End Products via mTOR/S6K1/LC3 Pathway in Diabetic Nephropathy
Source: Evid Based Complement Alternat Med. 2021 Aug 9;2021:9942678. doi: 10.1155/2021/9942678 (PMC8367537; doi:10.1155/2021/9942678)
Supplement: Supplementary Materials — Table S1: primers in this study. Table S2: the chemical composition of Yiqi Huoxue recipe under positive ion mode. Table S3: the chemical composition of Yiqi Huoxue recipe under negative ion mode. Figure S1: the workflow of this study. Figure S2: the total ion chromatogram of Yiqi Huoxue recipe detected by UHPLC-Q-TOF-MS. Figure S3: schematic diagram of TCM secondary spectrum library comparison with TCM MS/MS Library. Figure 4: the mTOR, S6K1, and LC3 II expression in the kidney tissues detected by qRT-PCR. [file 9942678.f1.zip › 9942678.f1/Supplemental Figure 2.pdf]

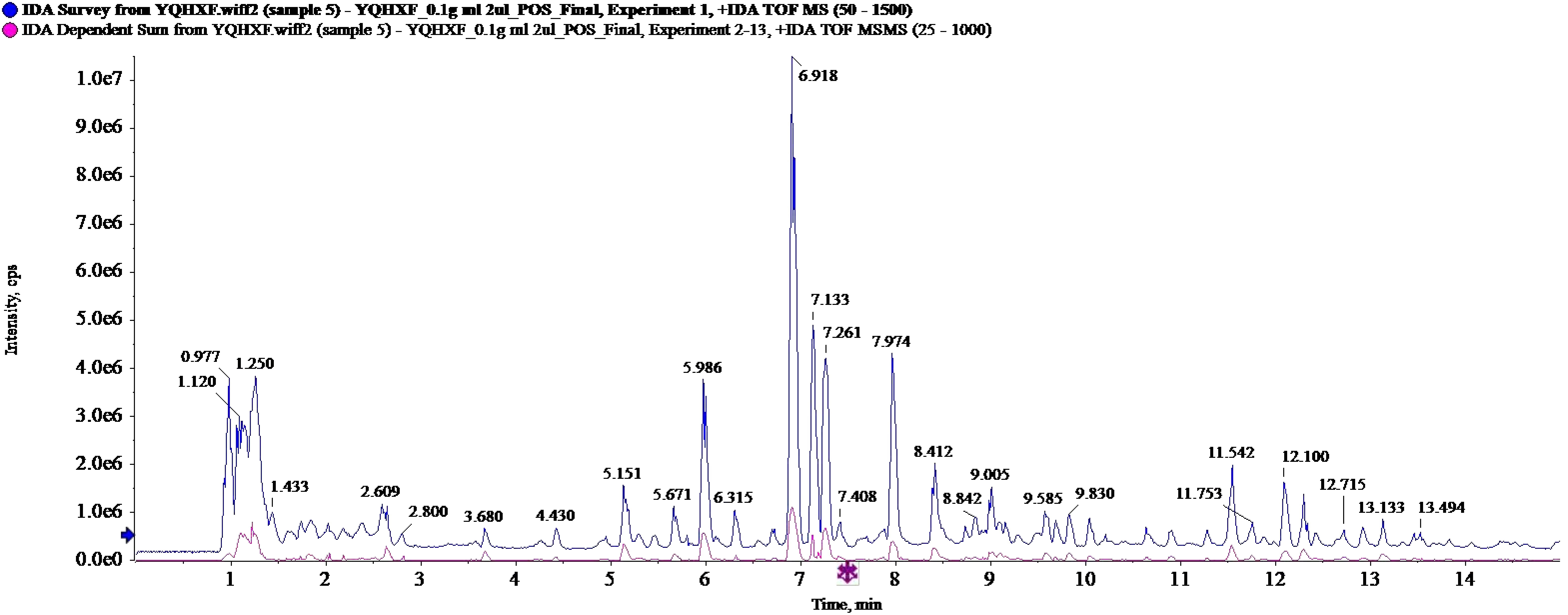
IDA Survey from YQHXF.wiff2 (sample 5) - YQHXF_0.1g ml 2ul_POS_Final, Experiment 1, +IDA TOF MS (50 - 1500)
IDA Dependent Sum from YQHXF.wiff2 (sample 5) - YQHXF_0.1g ml 2ul_POS_Final, Experiment 2-13, +IDA TOF MSMS (25 - 1000)
Intensity, cps
1.0e7
9.0e6
8.0e6
7.0e6
6.0e6
5.0e6
4.0e6
3.0e6
2.0e6
1.0e6
0.0e0
0.977
1.120
1.250
1.433
2.609
2.800
3.680
4.430
5.151
5.671
5.986
6.315
6.918
7.133
7.261
7.408
7.974
8.412
8.842
9.005
9.585
9.830
11.753
11.542
12.100
12.715
13.133
13.494
1
2
3
4
5
6
7
8
9
10
11
12
13
14
Time, min

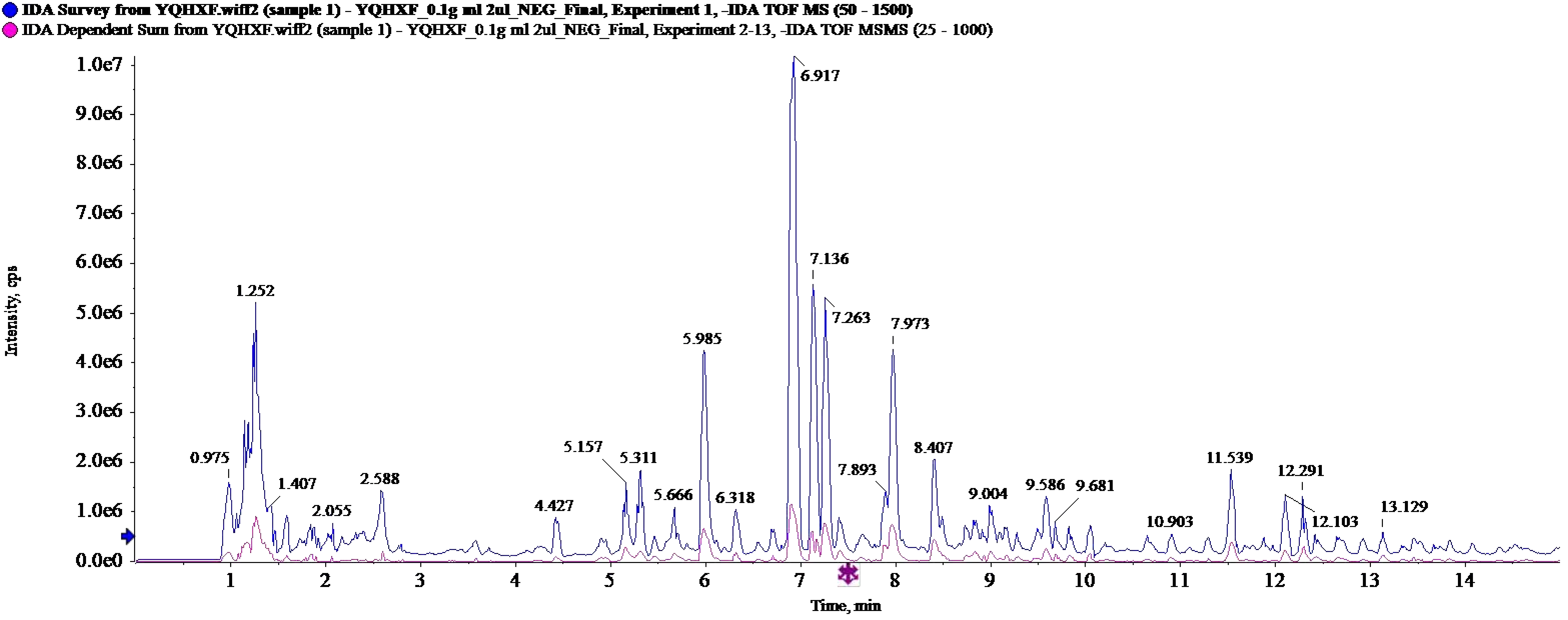
IDA Survey from YQHXF.wiff2 (sample 1) - YQHXF_0.1g ml 2ul_NEG_Final, Experiment 1, -IDA TOF MS (50 - 1500)
IDA Dependent Sum from YQHXF.wiff2 (sample 1) - YQHXF_0.1g ml 2ul_NEG_Final, Experiment 2-13, -IDA TOF MSMS (25 - 1000)
Intensity, cps
1.0e7
9.0e6
8.0e6
7.0e6
6.0e6
5.0e6
4.0e6
3.0e6
2.0e6
1.0e6
0.0e0
6.917
7.136
7.263
7.973
1.252
5.985
0.975
1.407
2.588
2.055
4.427
5.157
5.311
5.666
6.318
7.893
8.407
9.004
9.586
9.681
10.903
11.539
12.291
12.103
13.129
1
2
3
4
5
6
7
8
9
10
11
12
13
14
Time, min
