# Supplementary material for: Yiqi Huoxue Recipe Regulates Autophagy through Degradation of Advanced Glycation End Products via mTOR/S6K1/LC3 Pathway in Diabetic Nephropathy
Source: Evid Based Complement Alternat Med. 2021 Aug 9;2021:9942678. doi: 10.1155/2021/9942678 (PMC8367537; doi:10.1155/2021/9942678)
Supplement: Supplementary Materials — Table S1: primers in this study. Table S2: the chemical composition of Yiqi Huoxue recipe under positive ion mode. Table S3: the chemical composition of Yiqi Huoxue recipe under negative ion mode. Figure S1: the workflow of this study. Figure S2: the total ion chromatogram of Yiqi Huoxue recipe detected by UHPLC-Q-TOF-MS. Figure S3: schematic diagram of TCM secondary spectrum library comparison with TCM MS/MS Library. Figure 4: the mTOR, S6K1, and LC3 II expression in the kidney tissues detected by qRT-PCR. [file 9942678.f1.zip › 9942678.f1/Supplemental Figure 3.pdf]

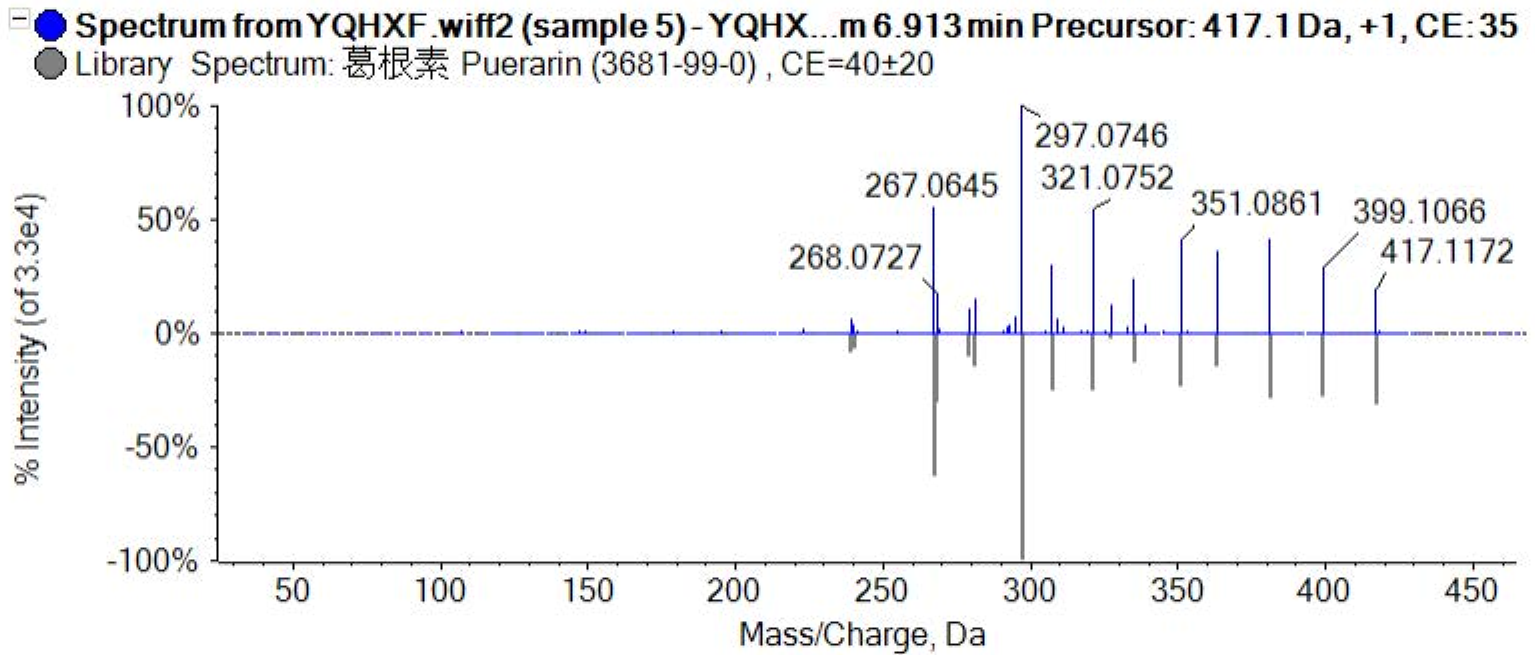
Spectrum from YQHXF.wiff2 (sample 5) - YQHX...m 6.913 min Precursor: 417.1 Da, +1, CE: 35
Library Spectrum: 葛根素 Puerarin (3681-99-0) , CE=40±20
100%
50%
0%
-50%
-100%
% Intensity (of 3.3e4)
297.0746
267.0645
321.0752
351.0861
399.1066
268.0727
417.1172
50
100
150
200
250
300
350
400
450
Mass/Charge, Da

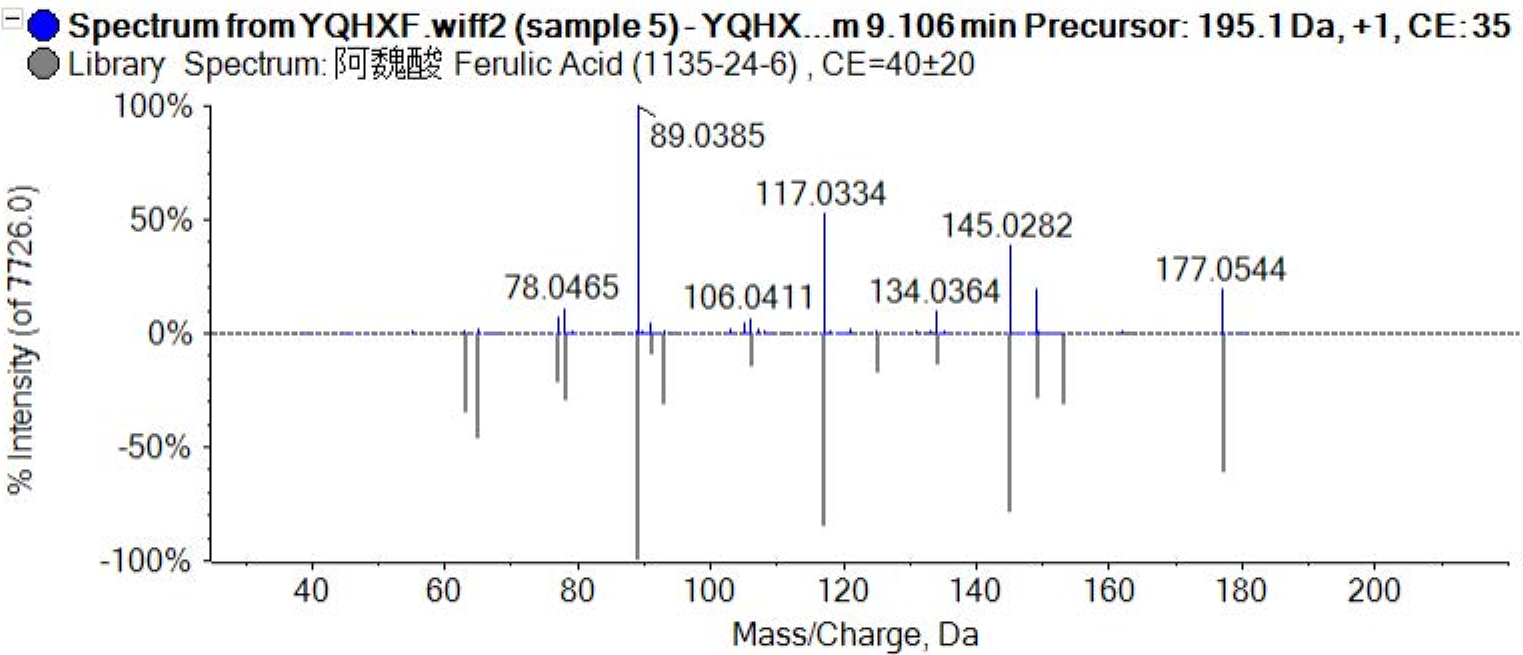

Spectrum from YQHXF.wiff2 (sample 5) - YQHX...m 9.106 min Precursor: 195.1 Da, +1, CE: 35
Library Spectrum: 阿魏酸 Ferulic Acid (1135-24-6) , CE=40±20
100%
50%
0%
-50%
-100%
% Intensity (of 7726.0)
89.0385
117.0334
145.0282
177.0544
78.0465
106.0411
134.0364
40
60
80
100
120
140
160
180
200
Mass/Charge, Da

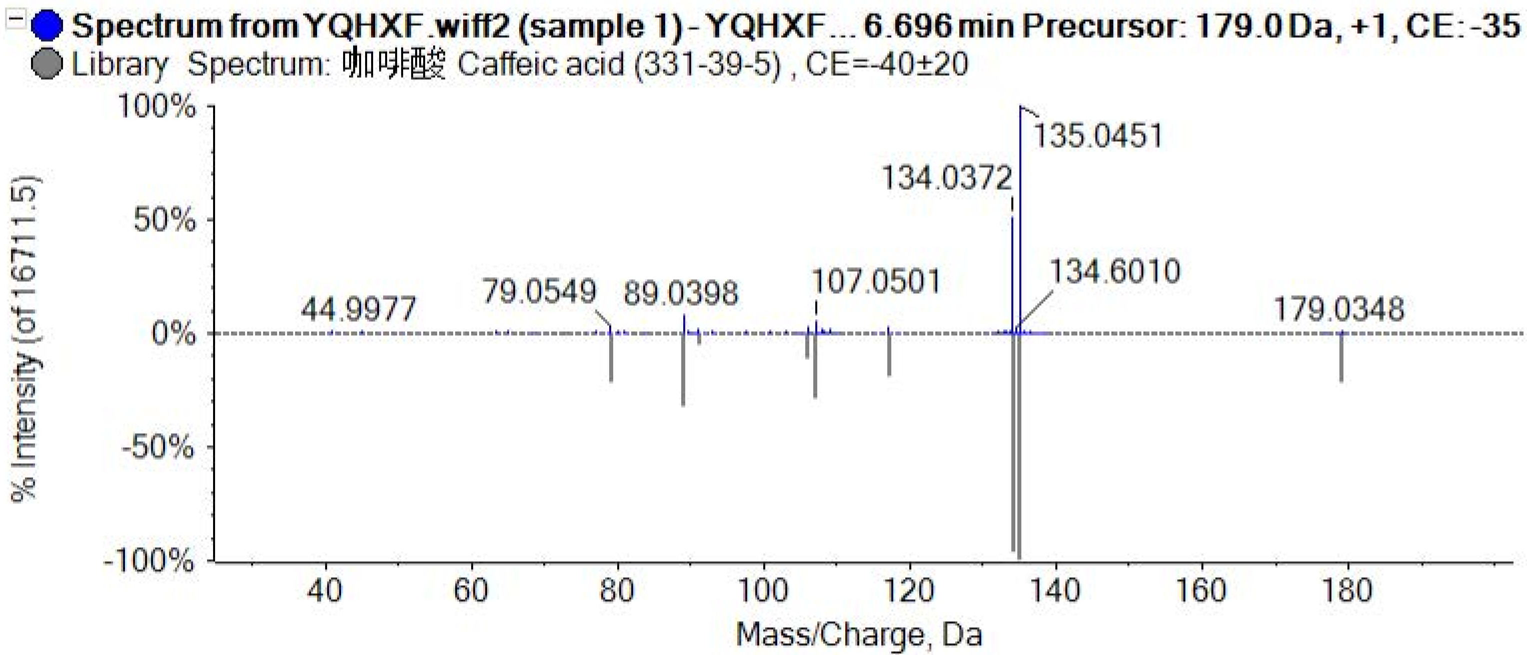

Spectrum from YQHXF.wiff2 (sample 1) - YQHXF... 6.696 min Precursor: 179.0 Da, +1, CE: -35
Library Spectrum: 咖啡酸 Caffeic acid (331-39-5) , CE=-40±20
135.0451
134.0372
134.6010
107.0501
89.0398
79.0549
44.9977
179.0348
100%
50%
0%
-50%
-100%
% Intensity (of 16711.5)
40
60
80
100
120
140
160
180
Mass/Charge, Da

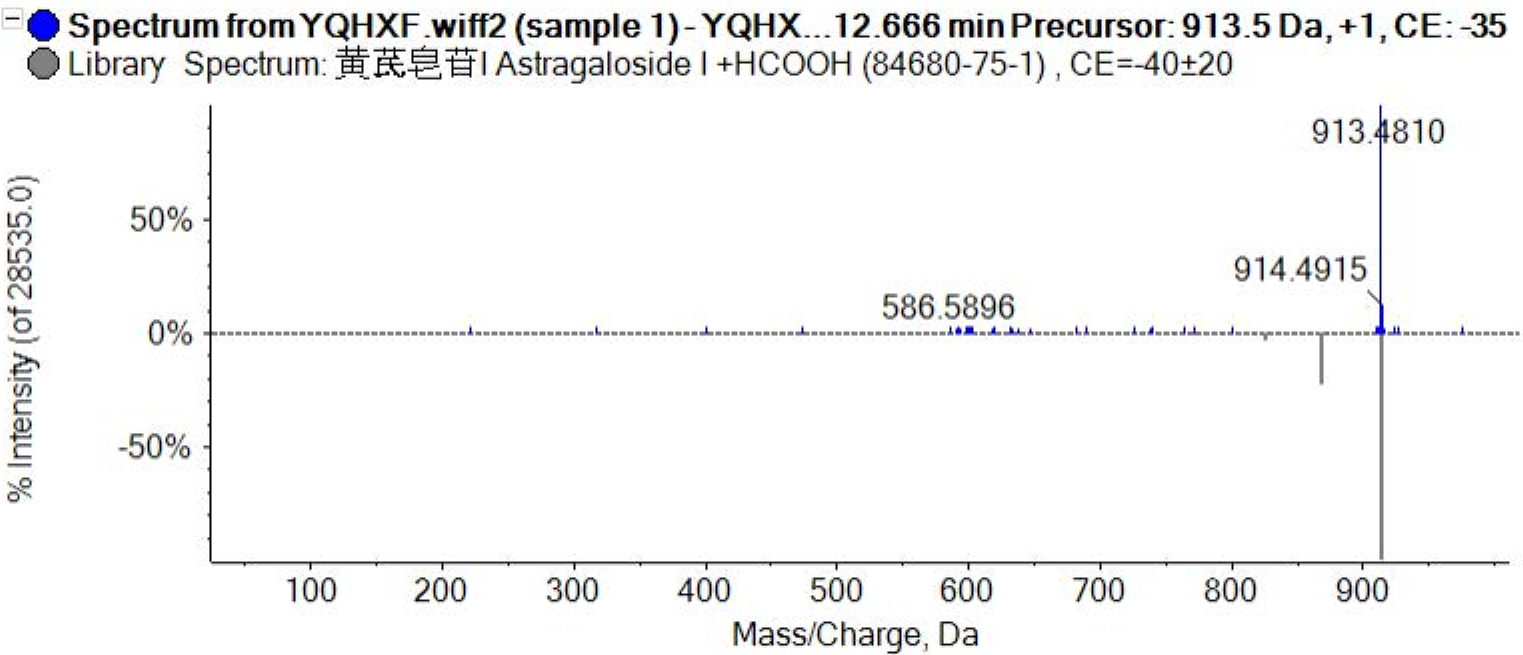

Spectrum from YQHXF.wiff2 (sample 1) - YQHX...12.666 min Precursor: 913.5 Da, +1, CE: -35
Library Spectrum: 黄芪皂苷I Astragaloside I +HCOOH (84680-75-1) , CE=-40±20
913.4810
914.4915
586.5896
50%
0%
-50%
% Intensity (of 28535.0)
100
200
300
400
500
600
700
800
900
Mass/Charge, Da
