# Supplementary material for: Yiqi Huoxue Recipe Regulates Autophagy through Degradation of Advanced Glycation End Products via mTOR/S6K1/LC3 Pathway in Diabetic Nephropathy
Source: Evid Based Complement Alternat Med. 2021 Aug 9;2021:9942678. doi: 10.1155/2021/9942678 (PMC8367537; doi:10.1155/2021/9942678)
Supplement: Supplementary Materials — Table S1: primers in this study. Table S2: the chemical composition of Yiqi Huoxue recipe under positive ion mode. Table S3: the chemical composition of Yiqi Huoxue recipe under negative ion mode. Figure S1: the workflow of this study. Figure S2: the total ion chromatogram of Yiqi Huoxue recipe detected by UHPLC-Q-TOF-MS. Figure S3: schematic diagram of TCM secondary spectrum library comparison with TCM MS/MS Library. Figure 4: the mTOR, S6K1, and LC3 II expression in the kidney tissues detected by qRT-PCR. [file 9942678.f1.zip › 9942678.f1/Supplemental Table 1.pdf]

Supplemental Table 1 Primers in this study

Gene	Forward Primer	Reverse Primer
mTOR	AGGCTTGATTCAGTTCCCAGG	GTTGAAGCCCCTGGTTGAGA
S6K1	CCTGGAATGCTTTCGATGGAG	CCTGGAATGCTTTCGATGGAG
LC3 II	CTCCCAAGAAACCTTCGGCT	GACTTGGTATGCTGGCTGGT
GAPDH	GATGGTGAAGGTCGGTGTGA	TGAACTTGCCGTGGGTAGAG
