# Supplementary material for: Yiqi Huoxue Recipe Regulates Autophagy through Degradation of Advanced Glycation End Products via mTOR/S6K1/LC3 Pathway in Diabetic Nephropathy
Source: Evid Based Complement Alternat Med. 2021 Aug 9;2021:9942678. doi: 10.1155/2021/9942678 (PMC8367537; doi:10.1155/2021/9942678)
Supplement: Supplementary Materials — Table S1: primers in this study. Table S2: the chemical composition of Yiqi Huoxue recipe under positive ion mode. Table S3: the chemical composition of Yiqi Huoxue recipe under negative ion mode. Figure S1: the workflow of this study. Figure S2: the total ion chromatogram of Yiqi Huoxue recipe detected by UHPLC-Q-TOF-MS. Figure S3: schematic diagram of TCM secondary spectrum library comparison with TCM MS/MS Library. Figure 4: the mTOR, S6K1, and LC3 II expression in the kidney tissues detected by qRT-PCR. [file 9942678.f1.zip › 9942678.f1/Supplemental Table 2.pdf]

Supplemental Table 2 The chemical composition of Yiqi Huoxue Recipe under positive ion mode

No.	Component Name	Area	Retention Time	Formula	Precursor Mass	Found At Mass	Mass Error (ppm)	Library Score	Isotope Ratio Difference
1	Arginine	3915000	1.1	$C_6H_{14}N_4O_2$	175.119	175.119	-1.1	93.3	2.2
2	Threonine	103700	1.13	$C_4H_9NO_3$	120.066	120.066	0.7	90.1	0.6
3	Glutamic acid	101700	1.15	$C_5H_9NO_4$	148.06	148.061	1	99.6	2.8
4	Citrulline	1095000	1.15	$C_6H_{13}N_3O_3$	176.103	176.103	0.1	98.4	3.3
5	Betaine	116400	1.16	$C_5H_{11}NO_2$	118.086	118.086	1.3	100	1
6	Trigonelline	1019000	1.2	$C_7H_7NO_2$	138.055	138.055	-1	95.2	1.2
7	Proline	3273000	1.23	$C_5H_9NO_2$	116.071	116.071	-0.7	97.6	0.6
8	Adenine	368100	1.67	$C_5H_5N_5$	136.062	136.062	-0.9	95.2	3.2
9	Pipecolinic acid	226000	1.73	$C_6H_{11}NO_2$	130.086	130.087	2.2	72.5	1.2
10	Nicotinic acid	164800	1.74	$C_6H_5NO_2$	124.039	124.039	-0.2	97.4	1.7
11	Nicotinamide	52490	1.87	$C_6H_6N_2O$	123.055	123.055	0.5	98.2	0.9
12	6-Hydroxypurine	27220	1.95	$C_5H_4N_4O$	137.046	137.046	-0.4	82.6	1.3
13	Adenosine	1390000	2.65	$C_{10}H_{13}N_5O_4$	268.104	268.104	-0.3	98	4.5

14	Cordycepin	22000	2.74	$C_{10}H_{13}N_5O_3$	252.109	252.109	0.2	100	2.8
15	Guanosine	247100	2.8	$C_{10}H_{13}N_5O_5$	284.099	284.099	0.1	100	4.5
16	Rehmannioside D +NH3	45980	3.58	$C_{27}H_{42}O_{20}.NH_3$	704.261	704.261	0.7	93.3	9.1
17	Phenylalanine	320300	3.68	$C_9H_{11}NO_2$	166.086	166.086	-0.6	99.8	3.8
18	Glucosylvitexin	506700	4.96	$C_{27}H_{30}O_{15}$	595.166	595.166	-0.2	97.1	11.2
19	4-Hydroxybenzoic acid	6157	5.81	$C_7H_6O_3$	139.039	139.039	0	78	4
20	Vitexin	9961000	5.99	$C_{21}H_{20}O_{10}$	433.113	433.113	-0.6	97.9	5.5
21	Chlorogenic acid	195400	6.05	$C_{16}H_{18}O_9$	355.102	355.102	-0.3	100	6.1
22	Vaccarin	48360	6.19	$C_{32}H_{38}O_{19}$	727.208	727.208	0.1	84	8.5
23	Puerarin	29770000	6.92	$C_{21}H_{20}O_9$	417.118	417.118	-0.9	98	7.6
24	Vitamin B2	10050	7.22	$C_{17}H_{20}N_4O_6$	377.146	377.145	-0.9	80.8	4.3
25	Anise oil	6941	7.38	$C_{10}H_{12}O$	149.096	149.096	-0.4	74.1	14.1
26	Echinacoside +NH3	33860	7.65	$C_{35}H_{46}O_{20}.NH_3$	804.292	804.292	-0.1	95	12
27	Ferulic Acid	167500	9.1	$C_{10}H_{10}O_4$	195.065	195.065	-0.4	77.1	2.3
28	8-hydroxy-6,7-dimethoxycoumarin	9054	9.36	$C_{11}H_{10}O_5$	223.06	223.06	1	85.3	0.1
29	IsoActeoside +NH3	9308	9.52	$C_{29}H_{36}O_{15}.NH_3$	642.239	642.239	-0.7	99.3	3.2
30	Genistein	275700	9.59	$C_{15}H_{10}O_5$	271.06	271.06	0.1	87.9	1.8

31	Naringenin	6809	10.46	$C_{15}H_{12}O_5$	273.076	273.076	0.2	95.7	5.6
32	Pratensein-7-O-glucoside	135400	10.64	$C_{22}H_{22}O_{11}$	463.123	463.124	0.6	85.7	9.3
33	Senkyunolide I	20850	11.75	$C_{12}H_{16}O_4$	225.112	225.112	-0.4	70.2	4
34	Ononin	3927000	12.1	$C_{22}H_{22}O_9$	431.134	431.133	-1	98.6	7.4
35	Daidzein	3139000	12.3	$C_{15}H_{10}O_4$	255.065	255.065	-0.1	98.2	4.4
36	3-Hydroxy-9,10-Dimethoxypterocarpan	513100	12.92	$C_{17}H_{16}O_5$	301.107	301.107	-0.4	88.8	7.7
37	Calycosin	1765000	13.14	$C_{16}H_{12}O_5$	285.076	285.075	-1.4	95.2	2.2
38	Isomucronulatol-7-O-glucoside	17840	13.34	$C_{23}H_{28}O_{10}$	465.176	465.176	0.7	81.7	7.6
39	Isomucronulatol	67190	13.34	$C_{17}H_{18}O_5$	303.123	303.123	0.4	87	5.7
