# Supplementary material for: Yiqi Huoxue Recipe Regulates Autophagy through Degradation of Advanced Glycation End Products via mTOR/S6K1/LC3 Pathway in Diabetic Nephropathy
Source: Evid Based Complement Alternat Med. 2021 Aug 9;2021:9942678. doi: 10.1155/2021/9942678 (PMC8367537; doi:10.1155/2021/9942678)
Supplement: Supplementary Materials — Table S1: primers in this study. Table S2: the chemical composition of Yiqi Huoxue recipe under positive ion mode. Table S3: the chemical composition of Yiqi Huoxue recipe under negative ion mode. Figure S1: the workflow of this study. Figure S2: the total ion chromatogram of Yiqi Huoxue recipe detected by UHPLC-Q-TOF-MS. Figure S3: schematic diagram of TCM secondary spectrum library comparison with TCM MS/MS Library. Figure 4: the mTOR, S6K1, and LC3 II expression in the kidney tissues detected by qRT-PCR. [file 9942678.f1.zip › 9942678.f1/Supplemental Table 3.pdf]

Supplemental Table 3 The chemical composition of Yiqi Huoxue Recipe under negative ion mode

NO.	Component Name	Area	Retention Time	Formula	Precursor Mass	Found At Mass	Mass Error (ppm)	Library Score	Isotope Ratio Difference
1	Histidine	32090	1.07	$C_6H_9N_3O_2$	154.062	154.062	-1.1	82.2	0.7
2	Arginine	111200	1.07	$C_6H_{14}N_4O_2$	173.104	173.1042	-1.1	88.5	4.3
3	Citrulline	42640	1.15	$C_6H_{13}N_3O_3$	174.088	174.0885	0.4	97.7	2.6
4	Sorbitol	69100	1.15	$C_6H_{14}O_6$	181.072	181.0716	-0.9	91.2	3.4
5	D-(+)-Glucose	208600	1.21	$C_6H_{12}O_6$	179.056	179.0562	0.2	71	2.8
6	Citric acid	3341000	1.85	$C_6H_8O_7$	191.02	191.0196	-0.9	98.4	2.3
7	Succinic acid	110100	2.43	$C_4H_6O_4$	117.019	117.0194	0.8	91.5	0.3
8	Adenosine	16160	2.65	$C_{10}H_{13}N_5O_4$	266.089	266.0892	-1	94.7	3.2
9	Guanosine	451400	2.8	$C_{10}H_{13}N_5O_5$	282.084	282.0841	-1	99.2	6.6
10	Rehmannioside D +HCOOH	695800	3.58	$C_{27}H_{42}O_{20}$.HCOOH	731.225	731.2245	-0.9	98.7	13.1
11	Phenprobamate	118900	3.69	$C_9H_{11}NO_2$	164.072	164.0715	-0.9	99.4	4.5
12	Geniposidic acid	247200	4.31	$C_{16}H_{22}O_{10}$	373.114	373.1137	-1	97.4	8.1
13	Neochlorogenic acid	1132000	4.9	$C_{16}H_{18}O_9$	353.088	353.0875	-0.9	97.6	8

14	8-Epiloganic acid	204600	5.13	$C_{16}H_{24}O_{10}$	375.13	375.1295	-0.4	98.4	5.7
15	L-Tryptophan	199000	5.14	$C_{11}H_{12}N_2O_2$	203.083	203.0824	-0.8	97.3	3.5
16	Vitexin	10600000	5.99	$C_{21}H_{20}O_{10}$	431.098	431.0982	-0.5	80.1	8.9
17	Quinic acid	333200	6.06	$C_7H_{12}O_6$	191.056	191.056	-0.3	96.2	2.9
18	Fraxin	8161	6.69	$C_{16}H_{18}O_{10}$	369.083	369.0825	-0.7	96.1	1.6
19	Caffeic acid	209200	6.7	$C_9H_8O_4$	179.035	179.0348	-1	72.9	2.4
20	Puerarin	37290000	6.92	$C_{21}H_{20}O_9$	415.103	415.1029	-1.3	98.2	9.8
21	3,5-Dicaffeoyl quinic acid	94140	7.49	$C_{25}H_{24}O_{12}$	515.119	515.119	-0.9	97.4	6
22	Echinacoside	297800	7.64	$C_{35}H_{46}O_{20}$	785.251	785.2498	-1.4	99.1	15.4
23	Daidzin +HCOOH	10400000	7.97	$C_{21}H_{20}O_9$.HCOOH	461.109	461.1083	-1.4	100	10
24	Calycosin-7-o-glucoside +HCOOH	2432000	9.01	$C_{22}H_{22}O_{10}$.HCOOH	491.119	491.1191	-0.9	99.3	11.6
25	Ferulic Acid	677400	9.1	$C_{10}H_{10}O_4$	193.051	193.0503	-1.8	91.4	4.7
26	Acteoside; Verbascoside; Kusaginin	201200	9.53	$C_{29}H_{36}O_{15}$	623.198	623.1976	-0.9	98.4	11.2
27	Diosmetin	22480	10.64	$C_{16}H_{12}O_6$	299.056	299.0559	-0.8	83	5.2
28	4-Hydroxybenzoic acid	87160	11.18	$C_7H_6O_3$	137.024	137.0243	-0.9	100	2.5
29	Ononin +HCOOH	3132000	12.1	$C_{22}H_{22}O_9$.HCOOH	475.125	475.1243	-0.7	99.2	10.9
30	Cistanoside D +HCOOH	1781	12.25	$C_{31}H_{40}O_{15}$.HCOOH	697.235	697.2318	-4.5	98.6	5.7

31	Daidzein	3526000	12.29	$C_{15}H_{10}O_4$	253.051	253.0505	-0.6	98	6.2
32	Isoliquiritigenin	29410	12.58	$C_{15}H_{12}O_4$	255.066	255.0662	-0.5	92.2	3.9
33	Astragaloside I +HCOOH	80910	12.64	$C_{45}H_{72}O_{16}.HCOOH$	913.48	913.4795	-0.8	92.9	15.6
34	Epimeredinoside A	72390	12.95	$C_{31}H_{40}O_{15}$	651.229	651.2289	-0.8	87.4	13.5
35	Calycosin	1475000	13.13	$C_{16}H_{12}O_5$	283.061	283.061	-0.6	98.1	7.6
36	Isomucronulatol-7-O-glucoside	364800	13.34	$C_{23}H_{28}O_{10}$	463.161	463.1605	-0.9	97	11.4
37	Formononetin	375200	13.54	$C_{16}H_{12}O_4$	267.066	267.066	-1	74.1	8
38	3-Hydroxy-9,10-Dimethoxypterocarpan	28120	14.06	$C_{17}H_{16}O_5$	299.092	299.092	-1.8	85.8	0.8
